# Childbirth and motherhood in women with motor disability due to a rare condition: an exploratory study

**DOI:** 10.1186/s13023-021-01810-8

**Published:** 2021-04-13

**Authors:** Marc Dommergues, Drina Candilis, Ludivine Becerra, Edith Thoueille, David Cohen, Sylvie Viaux-Savelon

**Affiliations:** 1 Department of Obstetrics and Gynecology, Hopital Pitié Salpêtrière APHP and Sorbonne Université, 83 Boulevard de l’Hopital, 75013 Paris, France; 2 Université Paris Diderot, Paris, France; 3 Service d’Aide à la Parentalité des Personnes en Situation de Handicap, Paris, France; 4 Child Psychiatry, Hopital Pitié Salpêtrière APHP and Sorbonne Université, Paris, France

**Keywords:** Rare disease, Disability, Motherhood, Motor impairment Tetraplegia Hemiplegia  Amputation, Birth, Parent-infant interaction and relationship, Child development

## Abstract

**Background:**

Rare diseases may result in motor impairment, which in turn may affect parenthood. Our purpose was to evaluate perinatal outcomes, parenting needs, mother-infant interactions and infant development in a set of volunteer women with motor impairment due to a rare disease. In a parenting support institution, we recruited a consecutive series of 22 volunteer pregnant women or young mothers, recorded perinatal outcomes, and followed mother-infant interaction and relationship and infant development up to 14 months postpartum. Cases with intellectual or psychic disability were not included.

**Results:**

There were 11 genetic diseases (2 Spinal Muscular Atrophy, 1 Charcot-Marie-Tooth, 1 autosomal dominants myopathy, 1 mitochondrial disease, 2 Elhers-Danlos, 1 Friedreich ataxia, 1 spinocerebellar ataxia, 1 tetrahydrobiopterine deficiency,1 Ectrodactyly), and 11 rare non-genetic conditions (2 spine tumors, 2 strokes, 1 juvenile chronic arthritis, 3 birth injuries, 1 inflammatory myopathy, 1 congenital amputation, and 1 traumatic amputation). These resulted in 10 impairments of four limbs, 4 impairments of both lower limbs, 7 unilateral impairments, and one distal tremor. Social deprivation Epices score, Cutrona social support scale, Edinburg Postnatal Depression scale, and Spielberger State/Trait Anxiety Inventory were unremarkable. Perinatal outcome: 4 gestational diabetes, 1 pre-eclampsia, 9 caesareans, 6 assisted and 7 spontaneous vaginal deliveries, 20 term live-births and 2 premature deliveries (35–36 weeks). Twelve women declared they were self-sufficient for daily activities; six declared they were self-sufficient to provide basic care to their baby. Distribution of the Brunet-Lezine child development score was normal. The parent-infant relationship global assessment scale (PIR-GAS) was well adapted in 2 cases, adapted in 8, perturbed in 7, significantly perturbed in 2, and distressed in 3 (mean 71.8; 95% CI 49.6–93.9). This was unrelated to any somatic or emotional characteristics of the participants. Coding interactive behavior revealed that infant engagement was lower and infant avoidance greater than in controls (*p* < 0.05).

**Conclusion:**

Infant development was normal, but mother-infant interactions were altered in half of the participants independently from the degree of motor impairment, underscoring the need for parenting support, even for parents who are self-sufficient in daily activities.

## Background

About 10% of women of childbearing age are living with a disability that impedes their daily activities [1–4]. Among them, due to improved medical care, a growing number of women affected with a rare disease now reach childbearing age and have the same well-founded desire to become a mother as other women. Their ability to provide a safe environment for their child may be questioned by their families or by professionals, including social workers, nurses, midwives, or doctors [5–8]. When impairment results from a rare condition, women may face puzzled healthcare providers, who fear severe complications might occur should their patients become pregnant. Besides, women with disabilities often fail to obtain financial support to help them gain independence as caregivers to their children. These difficulties partly result from the scarcity of data on pregnancy, parenthood, and children’s outcomes in women affected by a rare disease with a motor expression. Our goal was to provide a new insight in that matter. Therefore, we recruited volunteers attending our parenting support facility for disabled persons, who consented to report on their experience as parents, have their child’s development assessed, have their infant screened for withdrawal, and undergo assessment of mother-infant relationship and interaction.

## Methods

### Aim, design, setting

Exploratory observational study conducted in a parenting support facility dedicated to disabled parents and future parents.

### Characteristic of participants

Between March 2016 and August 2017, we recruited participants among pregnant women or young mothers referred for parenting support. We followed pregnant women at least 3 months and up to 12 months post-partum, and recorded their perinatal data prospectively. For participants included in the post-partum period, we recorded perinatal data retrospectively.

Potentially eligible women received verbal and written information regarding the study. Additional information was provided to those who expressed interest, together with their partner if applicable. They gave written consent to participate. A specific consent form was signed for authorizing video of mother–child interactions. When applicable, the participant’s partner or the co-parent signed a consent form for the collection of information on their child and the recording of videos.

Inclusion criteria were maternal age 18 or greater, being either pregnant > 14 weeks, or mother of a child < 1 year, having motor disability due to a rare condition, and written consent to enroll. In compliance with French laws, the absence of health insurance coverage precluded participation in the study. Further exclusion criteria were intellectual disability, psychiatric disorder, or lack of fluency in French. The inclusion visit took place either pre or postnatally, immediately before a planned parenting support visit.

One hundred and fifteen pregnant women with motor disabilities and/or rare conditions were eligible. Twenty-nine consented to participate. Two were excluded because of pre-existing psychiatric disorders. One was excluded because of mild intellectual disability. Four declined to be monitored following inclusion. Twenty-two women had a complete follow-up and formed our dataset. Of these, 15 consented to be videotaped with their infant.

### Processes and variables

At inclusion, data were collected based on medical records and self-administered questionnaires. The investigator helped the participant if she felt it difficult to handle the forms.

We recorded the following variables:

#### Motor impairment and disability

The name of the disease affecting the patient was recorded based on the participant’s declaration and on the specifications of her medical record. We used the Barthel index (BI) as a measure of functional independence in activities of daily living [9]. Participants declared if they felt independent, or needed help, when performing the following activities: feeding, bathing, dressing, bowel movements, bladder control, toilet use, transfers (bed to chair and back), and mobility. This resulted in a score ranging from zero (completely dependent) to 100 (completely independent). We did not modify scoring based on our own observations.

We also asked participants if they felt they were independent or needed help for each of the following parenting activities: changing or bathing, feeding, outing, dressing, playing, cuddling, and calming down a crying baby. For each activity, participants declared if they were independent (with or without an adapted environment), could do the activity with help, or were unable to do it. Eventually this enabled us to identify two groups of participants: those who considered they were independent for all baby-care activities and those who declared they needed help for at least one activity. We included a detailed description of the number of body parts with motor impairment and additional non-motor impairment.

#### Obstetrical and perinatal data

We recorded obstetrical history and pregnancy outcomes based on the declaration of participants and on their medical records.

#### Social context

Social context was assessed based on marital status, housing, source of income, education level, the Epices score of social deprivation [10, 11] and the Cutrona scale for social support [12, 13].

#### Emotional status

We assessed maternal emotional status at inclusion and during the postnatal visit. At inclusion, we used the State Trait Anxiety Inventory (STAI)-A for anxiety level, the STAI-B for anxiety trait [14–16]. At the post-natal visit we used the State Trait Anxiety Inventory (STAI)-A for anxiety level, and the Edinburg Postnatal Depression (EPDS) scale [17] for depressive mood. Participants were considered as depressed when EPDS score was greater than 12 [18, 19]. Anxiety was considered high when STAI score was greater than 42 [14].

#### Child development and mother-infant interaction

The postnatal visit took place between 3 and 13 months after delivery. One of the authors (LB, a clinical psychologist) made an appointment with the mother either at home (21 cases) or at the parenting support center (one case). She assessed child psychomotor development using the Brunet Lézine scale. This scale, designed for 2–30 month infants, analyses motor or postural development, eye-hand coordination, vocalization, and sociability. Combining these items results in a global developmental score [20, 21].

Infant withdrawal was assessed by LB using the Guedeney and Fermanian Modified Alarm Distress Baby Scale, M-ADBB [22–24]. Briefly, m-ADBB is a screening tool including only five areas: (a) facial expression, (b) eye contact, (c) vocalization, (d) activity level, and (e) relationship. In addition, the scoring is changed to three global levels: “Satisfactory,” “Possible problem,” or “Definite problem” for each area. “Definite problem” or two “Possible problems” on the m-ADBB indicates that the infant required further assessment.

The mother-infant relationship was assessed by LB based on a clinical evaluation using the parent-infant relationship global assessment scale (PIR-GAS) [25]. The PIR-GAS allows for a global rating of the quality of a parent-infant (or parent–child) relationship on a numerical scale, with higher scores indicating higher relationship quality. We used the original score, which classifies the quality of relationship as follows. 90: well adapted, 80: adapted, 70: perturbed, 60 significantly perturbed, 50: distressed, 40: disturbed, 30: disordered, 20: severely disordered, and 10: grossly impaired [26].

When parents gave consent, mother-infant interactions were videotaped. Mothers freely fed or played with their child either at home or at the parenting support center. Videos were analyzed offline by a trained child psychiatrist (SVS) blinded to the perinatal history, using the Coding Interactive Behavior (CIB) New-born and Feeding Scale [27, 28] using a validated French version [29]. The CIB is a global rating system of parent–child interaction that contains micro-level codes and global rating scales. Each code is rated from 1 (a little) to 5 (a lot). Forty-two different codes are grouped into several interactive composites. Six composites were used in the current study focusing on the mother (N = 2), the infant (N = 2) and the dyad (N = 2). (1) Maternal sensitivity was the average of maternal acknowledgment of infant interactive signals, imitation and elaboration of the infant’s behavior, gaze directed to the infant or joint activity, appropriate tone of voice/motherese, expression of appropriate range of affect, resourcefulness in dealing with infant negative states, affectionate touch, supportive presence, and infant-led interaction, i.e. mother focusing on the child’s needs and state. (2) Maternal intrusiveness was the average of maternal inappropriate physical manipulation, mother overriding behavior (i.e. mother disregarding the infant’s signals and interrupting the infant’s ongoing behavior), maternal anxiety, maternal negative affect/anger toward the baby, maternal criticizing of infant’s behavior, and mother-led interaction (i.e. interactions being led by the mother’s needs rather than infant’s needs, pace, and agenda). (3) Dyadic reciprocity was the average of the mother’s elaboration of the infant’s vocalizations and movements, maternal gaze directed to the infant, child gaze directed to mother or joint activity, verbal praise of the infant’s behavior, affectionate touch and enthusiasm, infant vocalization, warm and positive affect for both parent and child, dyadic adaptation—regulation, and fluency of the interaction. (4) Negative dyadic status was the average of maternal negative affect/anger, mother’s hostility behavior, child’s negative or labile affect, withdrawal of the infant from the environment, dyad constriction, and expression of tension. (5) Infant avoidance was the average of the child’s avoidance behavior toward the mother, child’s negative and labile affect, and withdrawal from the environment. (6) Infant social engagement was the average of joint attention, child positive affect, affection to parent, alertness, low fatigue, vocalizations/verbal output, initiation, competent use of the environment, and infant-led interaction.

During the post-partum visit, we also recorded somatic and psychological events that occurred before pregnancy, during pregnancy, and post-partum. We recorded the needs expressed by women regarding medical care, psychological, social, and environmental support (self-administered questionnaire). We recorded child protection legal decisions if applicable.

### Statistical analysis and data management

We used an electronic research form from Ad Scientiam, Paris, France (https://www.adscientiam.fr/). Data were stored anonymously, according to a procedure authorized by the *Comité Consultatif sur le traitement de l’information en matière de recherche* or CCTIRS, an independent agency of the French government. Most statistics were descriptive and exploratory. To compare the distribution of CIB dimensions to that of controls, we used videos from dyads enrolled as controls in a previous study [30]. We compared the median score for each dimension in the 15 cases who consented to video recordings to 13 controls using a bilateral Mann and Whitney non-parametric test. Statistics were run using StatView, Abacus Concept California USA.

## Results

### Mothers’ medical characteristics

Maternal age ranged from 26 to 41 years (mean 31,6 years). Ten women were included during pregnancy and 12 in the postnatal period. Eleven participants were primigravidae. Eleven had had a previous pregnancy, of which 7 had had 1, one had had 2, and three had had 3 previous pregnancies. Out of the 18 previous pregnancies, there were 7 spontaneous fetal losses < 24 weeks, two abortions for social reasons, 1 intrauterine death > 24 weeks, 1 premature live birth, and 7 term live births.

The type of motor impairment is displayed on Table 1. Associated disabilities and Barthel Indexes are displayed on Table 2.

**Table 1:** Motor impairment

Case number	Type of impairment	Movement disorders	Disease	Pathophysiology	Transmission	Risk for offspring
*Bilateral upper and lower limbs*						
1	Tetraparesis	No	Type 2 spinal muscular atrophy	Genetic	Autosomal recessive	Low
2	Tetraparesis	Yes	Type 2 spinal muscular atrophy	Genetic	Autosomal recessive	Low
3	Tetraparesis	Yes	Mitochondrial disorder	Genetic	Mitochondrial	Uncertain
4	Tetraparesis	Yes	Charcot Marie Tooth disease	Genetic	Autosomal recessive	Low
5	Tetraparesis	No	Autosomal dominant Myopathy	Genetic	Autosomal dominant	High
6	Tetraparesis	Yes	Cervical Hemangioma, C2-C3	Stroke	Not applicable	N/A
7	Bone/joints disorders	No	Juvenile chronic arthritis	Inflammatory	Not applicable	N/A
8	Bone/joints disorders	No	Ehlers-Danlos Syndrome	Genetic	Autosomal dominant	High
9	Bone/joints disorders	No	Ehlers-Danlos Syndrome	Genetic	Autosomal dominant	High
10	Ectrodactyly	No	Ectrodactyly of left upper limb, agenesis of right lower limb and left upper limb	Genetic	Possibly autosomal dominant	Uncertain
*Unilateral*						
11	Congenital Hemiplegia	Yes	Cerebral palsy	Birth asphyxia	Not applicable	N/A
12	Congenital Hemiplegia	Yes	Brain malformation	Malformation	Not applicable	N/A
13	Hemiparesis	Yes	Left hemiparesis	Stroke	Not applicable	N/A
14	Upper limb paresis	No	Brachial plexus birth injury	Birth injury	Not applicable	N/A
15	Upper limb paralysis	Yes	Brachial plexus birth injury	Birth injury	Not applicable	N/A
16	Amputation	No	Congenital agenesis of a forearm	Malformation	Not applicable	N/A
17	Amputation	No	Traumatic amputation of an arm	Trauma	Not applicable	N/A
*Bilateral lower limbs*						
18	Paraparesis	Yes	Friedreich’s ataxia	Genetic	Autosomal recessive	Low
19	Paraparesis	Yes	Spinocerebellar ataxia	Genetic	Autosomal dominant	High
20	Paraparesis	No	Autoimmune myopathy	inflammatory	Not applicable	Low
21	Brown-Sequard syndrome	No	Spinal astrocytoma	Tumor	Not applicable	Low
*Movement disorder*						
22	Distal tremor	yes	Tetrahydrobiopterin deficiency	Genetic	Autosomal recessive	Low

*N/A* not applicable

**Table 2:** Associated non-motor disabilities, Barthel index, and previous children

Case number	Dyspnea	Impaired vision	Impaired hearing	Hypoesthesia	Pain (past week)	Speech impairment	Barthel Index	Prev. children
1	Yes	No	No	No	4/10 and less	No	30	0
2	Yes	No	No	No	5/10 and less	No	30	0
3	No	No	No	No	4/10 and less	No	90	0
4	No	No	No	Yes	5/10 and less	No	60	1
5	No	No	No	No	4/10 and less	No	100	0
6	No	No	No	No	5/10 and less	No	95	0
7	Yes	No	No	No	4/10 and less	No	50	0
8	Yes	No	Yes	Yes	5/10 and less	No	70	0
9	Yes	No	No	Yes	4/10 and less	No	100	1
10	No	No	No	No	5/10 and less	No	85	0
11	No	No	Yes	Yes	5/10 and less	No	60	2
12	No	No	No	No	4/10 and less	Yes	75	0
13	No	Yes	No	Yes	5/10 and less	Yes	95	2
14	No	No	No	Yes	4/10 and less	No	100	0
15	No	No	No	Yes	5/10 and less	No	90	0
16	No	No	No	No	5/10 and less	No	100	0
17	No	No	No	Yes	5/10 and less	No	100	0
18	No	No	Yes	No	4/10 and less	Yes	55	0
19	No	Yes	Yes	No	4/10 and less	Yes	90	0
20	No	No	No	No	4/10 and less	No	85	0
21	No	No	No	Yes	5/10 and less	No	95	2

Regarding emotional status at inclusion the mean Spielberger YB trait score was 42.6 (SD 9.7). It was greater than 42 in 9 cases (range 50–68). The mean Spielberger YA state score was 41.1 (SD 15.2). It was greater than 42 in 10 cases (range 48–61).

At the post-natal visit, the mean EPDS score was 6.2 (SD 4.2). It was greater than 12 in two cases (13 and 16) and greater than 10 in three. The mean Spielberger YA state score was 34.7 (SD 12.0). It was greater than 42 in 5 cases (range 44–68).

### Social context

Twenty-one participants lived with a husband or partner. One was single. Six worked full time and 4 part-time, 3 were unemployed, 1 was a student, and 8 declared they were housewives. Seventeen of their partners worked full time and four were unemployed. One partner had epidermolysis bullosa. The others did not suffer from any chronic disease. Fifteen participants received social benefits. Twenty lived in their own home, and two lived at the home of a relative. Fifteen considered their home was adapted to their disability, and 7 declared it was not.

Educational level was as follows: graduated after three years or more of university (n = 11), graduated after two years of university, which is grossly equivalent to college graduation in the USA (n = 3), passed the final high school examination but did not attend university (n = 4), did not pass the final high school examination (n = 4).

Regarding social deprivation, 7/22 (32%) had an Epices score above 30, i.e. belonged to the quintiles 4 and 5 of social deprivation in France (4). The mean Cutrona social support score was 77 (SEM 1.9; SD 9.0), similar to what is expected in the general population (7).

### Obstetrical history and perinatal outcome

Twenty-one pregnancies were spontaneous, and one resulted from intrauterine insemination. Nineteen pregnancies were planned; three were unplanned, yet welcome.

Regarding obstetrical complications, there were 4 gestational diabetes, one pre-eclampsia. There were 9 caesareans, 6 assisted vaginal deliveries, and 7 spontaneous vaginal deliveries. Epidural or spinal anesthesia was provided in 16 cases, 4 women labored under opioids, and two caesarean sections were performed under general anesthesia.

Participants gave birth to 13 girls and 9 boys. Eight were bottle-fed, 14 were breast-fed. There were 20 live births at term and 2 premature deliveries at 35 and 36 weeks. One neonate required resuscitation maneuvers in the labor ward. Twenty-one did not: two had 5-min Apgar scores at 9 and nineteen at 10. Four neonates were transferred to the NICU (2 respiratory distresses, 1 hypoglycemia, 1 cephalhematoma). All babies were eventually alive and well at discharge. They all lived with their mothers. No legal measures were taken for child protection.

### Child development and mother-infant interaction

We performed the post-natal visit at three months in 10 cases, at four months in two, at eleven months in two, and at twelve months in 8.

**Fig. 1:**
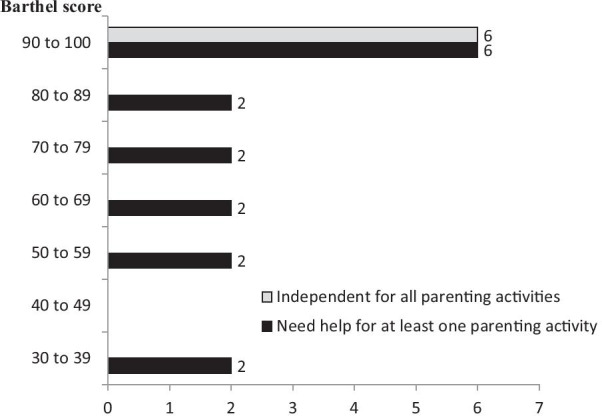
Barthel index and independence for parenting activities. *Note:* All women with Barthel score < 90 (i.e. dependent for daily activity) felt dependent for at least one parenting activity. Six of 12 women with Barthel score ≥ 90 (i.e. independent or nearly independent for daily activity) felt dependent for at least one parenting activity

As for mothers’ self-perception regarding independence for baby care activities, 21 participants declared they felt independent when playing with their baby, 20 when cuddling, 19 when feeding, 19 when calming the baby, 15 when dressing it, 12 when taking it on outings, and 10 when performing body care such as changing, cleaning or bathing the baby. Only 6 participants declared they considered themselves as independent during all the above activities. Declared independence regarding baby care activities did not correlate with the reported functional independence for self-care as assessed by the Barthel Index (Fig. 1).

**Fig. 2:**
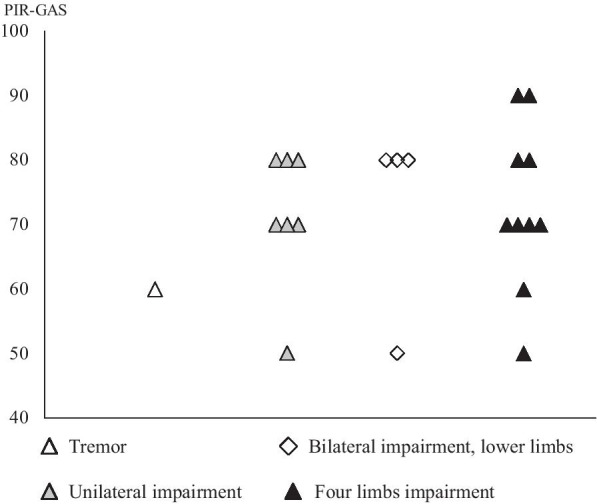
PIR-GAS and motor impairment. *Note:* Parent-Infant Global Assessment Score (PIR-GAS) was independent from the type of motor impairement

Child development as assessed by the distribution of the Brunet Lezine score was within normal range for all infants (mean, 99.8; range 91–109) Regarding the M-ADBB withdrawal scale, no infant was considered as having a “definite problem” and 5/22 (22%) were considered as having two “possible problems”, i.e. were positive for withdrawal screening. Based on PIR-GAS scoring, the mother-infant relationship was considered well adapted in 2 cases, adapted in 8, perturbed in 7, significantly perturbed in 2, and distressed in 3, with a mean score of 71.8 (95% confidence interval: 49.6–93.9). The five infants positive for M-ADBB withdrawal screening belonged to dyads with perturbed (n = 2), significantly perturbed (n = 2) or distressed (n = 1) mother-infant relationships assessed by the PIR-GAS score. PIR-GAS scores were not correlated to the degree of motor impairment (Fig. 2), to the independence women perceived in their activities of daily living (Bathel index), to the age at which the disease started, to the indices of social support or of social deprivation, or to the scores of anxiety and depression (data not shown).

**Fig. 3:**
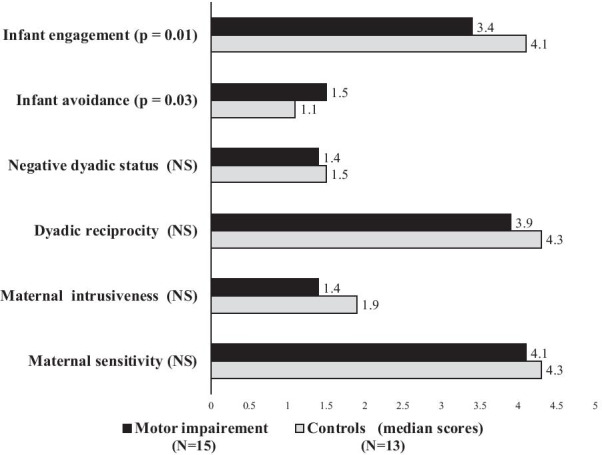
Coding interactive behavior: cases vs. controls. *Note:* Mother-infant interactions were significantly different regarding infant engagement towards the mother and infant avoidance of the mother in dyads with maternal motor disability compared to control dyads without maternal disability

Finally, Fig. 3 displays the distribution of CIB scores in participants compared to controls. The median score for infant engagement was significantly lower and the median score for infant avoidance was significantly greater in dyads with maternal motor impairment than in control ones.

## Discussion

Our results suggest that in selected cases, with dedicated perinatal and parenting support, women with motor impairment resulting from a rare condition can achieve a successful pregnancy with a good short-term outcome in terms of infant development and health, and a reasonably good outcome in terms of mother-infant interactions. There was no correlation between the quality of mother-infant interactions and the degree of motor impairment. In addition, independence for activities of daily living did not predict independence for baby care. Half of the women who considered themselves as nearly or completely independent in daily activities declared that they were not when performing at least one parenting activity. All women with dependency in daily activity also felt dependent in parenting.

To the best of our knowledge, our exploratory study is the first to provide insight into child development and mother-infant interactions in women with motor disabilities due to a rare condition. Previous studies focused on fecundity [2, 31, 32], health care consumption [1, 33], the risk of post-partum depression [34], somatic perinatal and maternal outcomes [35–38], women’s expectations [3, 5, 39], and the opinion of professional [36, 40, 41] or lay people [7, 42].

Consequently, to date, recommendations have been issued mainly based on the opinions of experts [36, 41]. In an attempt to assess whether the environment provided by disabled mothers could lead to normal infant development, and safe mother- infant interactions, we focussed on infant development and mother–child interactions using a comprehensive clinical approach.

Our study is only exploratory given its numerous limitations. First, we failed to follow our initial plan, which was to use women with traumatic spine injury as a comparison group with women having a rare disease. We failed to recruit such pregnant women, because one of the obstetrical teams that was to be part of the project withdrew before we started inclusions. This made it impossible for us to determine the specific role of the etiology of the motor deficiency on parenthood. In our study, the conditions leading to motor disability were very heterogeneous and so were the degree and type of impairment. This is an obvious limitation in terms of generalization. The wide spectrum of disabilities represented, however, enabled us to show that the mother-infant interactions did not parallel the severity of motor dysfunctions. This is, in our view, a substantial result: one should not assume that interactions will go wrong on the basis that the mother has a severe motor impairment.

Second, our sample size is small, and self-selection of participants hampers generalization of our results. It is possible that mothers with severe social or parenting problems were not willing to participate. The professionals who offered women to participate also hypothesized that participants might have had a greater medical literacy than those who declined. This feeling, however, was not based on facts, since we did not ask potentially eligible persons why they choose to participate or not. Among participants, social support and social deprivation were similar to what is expected in the French population. The proportion of participants who had passed the final secondary education examination was slightly greater (81.8%) than what was found in 2018 in France, i.e. 71.9 and 69.5%, among women aged 25–34 and 35–44 respectively. We reckon that the sample we analyzed is not representative of a hypothetical reference population of women with a motor disability. Indeed our goal was to provide an exploratory insight on the mechanism of parenting in young mothers with motor impairment, not to draw general conclusions on their parenting skills.

Evaluating obstetrical outcomes was not the main goal of our study. The numbers are too small and the conditions too heterogeneous to allow for a precise obstetrical interpretation. The main point however, is that there was no life-threatening maternal or neonatal complication, despite the fact that the rate of gestational diabetes (18%), caesarean (41%), prematurity (9%) were greater than what is expected in the general population in France, i.e. respectively: 11%, 20%, 7%. The rates of epidural analgesia and the rate of assisted vaginal delivery were unremarkable.

Generalization of our results should also take into account the fact that participants received intense pre- and post-natal parenting support from a dedicated institution, which is likely to have gradually optimized care since one of the authors (ET) founded it in the mid 1980′s.

Interpreting our results in terms of outcome is complex. The time span over which we assessed infant development and infant-mother interactions is not an issue, since each measurement took into account the baby’s age. Short-term child development was unarguably standard. The rate of PIR-GAS scoring considered, perturbed or distressed was slightly above 50%. This is more than expected in the general population; i.e. around 20–30% [43], but slightly less than what has been found in dyads following a premature delivery [44]. The fact that no PIR-GAS score was below 50 is reassuring, suggesting there was no case of child neglect. However, CIB assessment evidenced that infants are aware of their mothers’ difficulties. Despite normal CIB maternal and dyadic scores, we found that the median infant avoidance score was greater and the infant engagement lower in dyads with maternal motor impairment than in controls. These differences are mild, yet statistically significant. Their magnitude is not greater than what we observed in women with no impairment who had a normal baby, but had been exposed to the prenatal finding of a minor variant at fetal screening ultrasound [30]. These findings support the idea that parenting support should be continued after the first year of the infant.

The very presence of the disability may have hampered the evaluation of interactions. For example, hyperkinetic movement disorders such as tremor or ataxia, present in 3 out of 22 participants, could possibly give a false impression of heightened intensity of emotion. The assessor was aware of such confounding effects, and was able to discern them with reasonable confidence.

Interestingly, low PIR-GAS scores were not correlated with the degree of motor impairment, the independence women perceived in their daily life, the age at which the disease started, the indices of social support or of social deprivation, or to the scores of anxiety and depression. This suggests that parenting is a complex process whose success cannot be anticipated by gross indicators such as motor impairment. It is also possible that we could not predict PIR-GAS based on maternal characteristics because factors we did not study played a key role, for example the situation of the co-parent and the family at large.

The fact that declared independence for baby care did not match with declared independence in activities of daily living assessed by the Barthel index was striking. This suggests that disability in parenting activity should be compensated for specifically, even in persons who otherwise declare they achieve independence in daily activities. What would be the key points to meet the needs of mothers with disabilities? Based on our clinical experience more so than on the results of this study, we believe the process could be as follows: (1) Explore what is needed to safely provide for the infant’s basic needs, based on prenatal training, including simulation exercises. (2) For each specific activity e.g. handling, bathing, changing the baby, provide adaptations of the environment and human support needed for the baby and the parents to feel safe. In the most severe cases, such as tetraplegia, this may require to hire a person dedicated to helping the mother cope with her infant 24 h a day. We believe society should bear the costs related to parenting support in addition to those dedicated to activities of daily living.

In conclusion, our results support the hypothesis that at least in the short run and with extensive support, some women with motor deficiencies resulting from rare conditions may become successful parents. Parenting difficulties may occur, however, underlying the need for support based on what mothers express and on the clinical evaluation of the mother-infant interaction. In the future, larger surveys analyzing simpler outcome criteria are required to provide a wider picture of parenting skills and needs when parents have motor disabilities.

## Data Availability

The datasets generated during and/or analysed during the current study are available from the corresponding author on reasonable request.
